# Association of *MTHFR *gene polymorphisms with breast cancer survival

**DOI:** 10.1186/1471-2407-6-257

**Published:** 2006-10-27

**Authors:** Damali N Martin, Brenda J Boersma, Tiffany M Howe, Julie E Goodman, Leah E Mechanic, Stephen J Chanock, Stefan Ambs

**Affiliations:** 1 Laboratory of Human Carcinogenesis, Center of Cancer Research (CCR), National Cancer Institute (NCI), National Institutes of Health (NIH), Bethesda, MD, USA; 2 Cancer Prevention Fellowship Program, Division of Cancer Prevention, NCI, NIH, Bethesda, MD, USA; 3 Gradient Corporation, Cambridge, MA, USA; 4 Section on Genomic Variation, Pediatric Oncology Branch, CCR, NCI, NIH, Bethesda, MD, USA

## Abstract

**Background:**

Two functional single nucleotide polymorphisms (SNPs) in the 5,10-methylenetetrahydrofolate reductase (*MTHFR*) gene, C677T and A1298C, lead to decreased enzyme activity and affect chemosensitivity of tumor cells. We investigated whether these *MTHFR *SNPs were associated with breast cancer survival in African-American and Caucasian women.

**Methods:**

African-American (n = 143) and Caucasian (n = 105) women, who had incident breast cancer with surgery, were recruited between 1993 and 2003 from the greater Baltimore area, Maryland, USA. Kaplan-Meier survival and multivariate Cox proportional hazards regression analyses were used to examine the relationship between *MTHFR *SNPs and disease-specific survival.

**Results:**

We observed opposite effects of the *MTHFR *polymorphisms A1298C and C677T on breast cancer survival. Carriers of the variant allele at codon 1298 (A/C or C/C) had reduced survival when compared to homozygous carriers of the common A allele [Hazard ratio (HR) = 2.05; 95% confidence interval (CI), 1.05–4.00]. In contrast, breast cancer patients with the variant allele at codon 677 (C/T or T/T) had improved survival, albeit not statistically significant, when compared to individuals with the common C/C genotype (HR = 0.65; 95% CI, 0.31–1.35). The effects were stronger in patients with estrogen receptor-negative tumors (HR = 2.70; 95% CI, 1.17–6.23 for A/C or C/C versus A/A at codon 1298; HR = 0.36; 95% CI, 0.12–1.04 for C/T or T/T versus C/C at codon 677). Interactions between the two *MTHFR *genotypes and race/ethnicity on breast cancer survival were also observed (A1298C, *p*_*interaction *_= 0.088; C677T, *p*_*interaction *_= 0.026).

**Conclusion:**

We found that the *MTHFR *SNPs, C677T and A1298C, were associated with breast cancer survival. The variant alleles had opposite effects on disease outcome in the study population. Race/ethnicity modified the association between the two SNPs and breast cancer survival.

## Background

Epidemiologic evidence shows that folate deficiency is a breast cancer risk factor [1,2]. MTHFR is a key enzyme in the folate metabolism pathway and regulates the intracellular folate pool for synthesis and methylation of DNA [3,4]. Two common allele variants of the *MTHFR *gene have been described, C677T (NCBI SNP ID: rs1801133) and A1298C (rs1801131), that lead to amino acid substitutions, Ala222Val and Glu429Ala, and to decreased enzyme activity [5-7]. Heterozygous and homozygous carriers of the 677T allele variant have a 30–40% and 60–70% reduced enzyme activity, respectively, as determined by *in vitro *analysis of the MTHFR activity [5,7,8]. The effect of the 1298C allele variant is less severe and homozygous carriers of this allele have a more moderate 30–40% reduction of the enzyme activity, yet its function remains controversial [6,7,9]. Furthermore, people who are heterozygous at both loci, C677T and A1298C, experience an intermediate activity loss of 40–50% [6,7]. It has been shown that the 677T variant increases the plasma homocysteine concentration in humans and reduces DNA methylation in cancer patients, which indicates a reduced synthesis of methionine and a more limited availability of the methyl donor, S-adenosyl-methionine, in the presence of the low activity T allele [5,10]. Many studies investigated the association between the two genotypes and breast cancer incidence. Although significant associations were observed in some studies, a clear linkage between *MTHFR *gene polymorphisms and the risk to develop breast cancer has not been established [11-18].

Recently, the two *MTHFR *genotypes were found to modulate the chemosensitivity of cancer cells to 5-fluorouracil and methotrexate [8,19]. Furthermore, the 677T variant has been linked to severe toxicity during adjuvant treatment of breast cancer with cyclophosphamide, methotrexate, and 5-fluorouracil (CMF) [20]. Other reports observed differing effects of the two genotypes on methotrexate efficacy and toxicity in patients with rheumatoid arthritis [21-23]. We hypothesized that the two SNPs may affect outcome of breast cancer patients with surgery and chemotherapy and studied the individual and combined effects of the SNPs on breast cancer survival. Because the tumor estrogen receptor-α (ER) status, race/ethnicity, and alcohol consumption have been shown to influence the metabolism of folate [1,2,17,24-28], we also examined possible interactions between these factors and the two *MTHFR *SNPs on breast cancer survival.

## Methods

### Study population

Breast cancer cases (n = 248) were women, who were recruited between February of 1993 and August of 2003 in the greater Baltimore area, as described previously [29]. All patients were identified through surgery lists and enrolled into the study prior to surgery. They were selected from a group of 361 breast cancer patients who were recruited at the University of Maryland Medical Center, the Baltimore Veterans Affairs Medical Center, Union Memorial Hospital, Mercy Medical Center, and the Sinai Hospital in Baltimore, Maryland. Selection was based on eligibility criteria and the availability of survival information. Cases were eligible if they had pathologically confirmed breast cancer, had residency in the greater Baltimore area, were of African-American or Caucasian descent by self-report, were diagnosed with breast cancer within the last six months prior to recruitment, and had, by self-report, no previous history of the disease. Patients were excluded if they were HIV, HCV, or HBV carriers, were IV-drug users, were institutionalized, or were physically or mentally unable to sign consent and complete the questionnaire. All participants signed a consent form and completed an interviewer-administered questionnaire. The blood collection was performed at the subjects' convenience but mostly at the conclusion of the interview. Of the patients that were eligible under the original protocol and could be contacted by phone, 83% agreed to participate in the study. One eligible patient died prior to surgery and is not included in the study population. Additional information to determine the ER status, disease stage, treatment, and survival was obtained from medical records and pathology reports, the Social Security Death Index, and the National Death Index. The Institutional Review Boards at the participating institutions approved the study.

### Histology evaluation and genotyping

A pathologist reviewed each tumor specimen to confirm the presence of tumor and the histology. Disease staging was performed according to the tumor-node-metastasis (TNM) system of the American Joint Committee on Cancer/the Union Internationale Contre le Cancer (AJCC/UICC). The two germline *MTHFR *SNPs, C677T and A1298C, were genotyped at the NCI Genotyping Core Facility (GCF), using the assay conditions described in the SNP500Cancer database [30]. We genotyped genomic DNA from fresh-frozen breast tissue (170 non-tumor tissues and 28 tumors) and buffy coat (n = 50) of the 248 patients. All genotype assays contained negative and positive controls, and 10% blinded duplicates consisting of patient samples. We successfully genotyped 98% of the cases for both genotypes (n = 243) and had a 100% concordance among blinded duplicates for the two assays. In addition, we analyzed the *MTHFR *C677T genotype of microdissected normal tissue from 21 of those 28 bulk tumor samples that were genotyped at the NCI GCF. The *MTHFR *genotype of these samples was determined by direct sequencing. We had 100% concordance between the C677T genotypes of the bulk tumor samples and the non-tumor tissue from these samples. The observed genotype frequencies for both SNPs were in Hardy-Weinberg equilibrium in our case population, and for a matched control population (n = 317). Genotype frequencies among African-Americans and Caucasians were in agreement with the frequency data for these race/ethnic groups in the SNP500Cancer database [30].

### Statistical analysis

Stata 7.0 (Stata Corp, College Station, TX) and SAS 8.0 (SAS Institute, Cary, NC) statistical softwares were used for data analysis. All statistical tests were two-sided and an association was considered statistically significant with *p *< 0.05. The Kaplan-Meier method and the log-rank test were used for univariate survival analysis. The Cox Proportional-Hazards regression was used for multivariate survival analysis to calculate adjusted hazard ratios. A statistical test for interaction was performed in Stata to determine if the effect of the two *MTHFR *genotypes on breast cancer survival is modified by other factors. In the analysis, an interaction on breast cancer survival was coded as "xi: stcox i.Var1*Var2". The interaction term was coded with *MTHFR *genotypes as Var1 and other factors (e.g., race, alcohol, and smoking) as Var2. Var1 and Var2 were coded as binary variables. For this test, the result was coded as zero (or referent) for the common MTHFR genotype (C/C for SNP677 and A/A for SNP1298) and one for the variant genotype (C/T or T/T for SNP677; A/C or C/C for SNP1298). Alcohol consumption was coded as "no/yes" for this test with "no" as the referent indicating a consumption of 12 or less alcoholic drinks in life at the time of recruitment. Survival was determined for the period from the date of hospital admission to the date of the last completed search for death entries in the Social Security Index (August 18, 2004). The median and mean follow-up times for overall survival were 47 and 55 months, respectively, with a range of 12 to 140 months for the follow up time. A total of 59 out of the 248 patients in the study (24%) died during this period. We obtained death certificates of the deceased cases and censored all causes of death that were not related to breast cancer, such as accidents, violent crimes, stroke, heart attack, and liver cirrhosis, in our analysis. The cause of death was not related to breast cancer for 5 patients.

## Results

### Study population and *MTHFR *genotype distribution

We studied the relationship between the *MTHFR *C677T and A1298C genotypes and disease outcome of 248 unselected breast cancer patients. The clinicopathological features of the patients are shown in Table 1. Most women were of African-American descent, and 132 patients received chemotherapy. Among those, 15 patients received a 5-fluorouracil/methotrexate-based therapy, 71 patients received a doxorubicin-based combination therapy, and the remaining 46 patients received either other agents or the exact treatment modality was not reported. The genotype distribution of the patients is shown in Table 2. The genotype frequencies for C677T and A1298C were significantly different between African-American and Caucasian women. We also observed a linkage between the two genotypes in our population (Table 3) that was consistent with published data [31,32]. None of the patients was homozygous for both variant alleles, and only two patients were homozygous for one variant allele and heterozygous for the other variant allele.

### MTHFR genotypes and breast cancer survival

**Figure 1:**
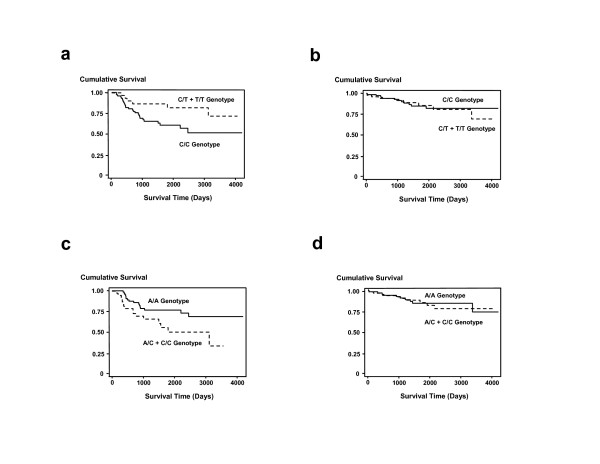
Association between *MTHFR *genotypes and breast cancer survival after stratification by ER-status. Shown are the Kaplan-Meier survival curves. (*a*) Cumulative survival of ER-negative patients by C677T status (n = 101). The survival of patients who carry the variant C677T genotype (n = 30; C/T or T/T) is significantly better than the survival of patients with the common genotype (n = 71; C/C). Log-rank test: *p *= 0.04. (*b*) Cumulative survival of ER-positive patients by C677T status (n = 144). The survival of patients who carry the variant C677T genotype (n = 50) is not significantly different from the survival of patients with the common genotype (n = 94). Log-rank test: *p *= 0.95. (*c*) Cumulative survival of ER-negative patients by A1298C status (n = 101). The survival of patients who carry the variant A1298C genotype (n = 38; A/C or C/C) is significantly worse than the survival of patients with the variant genotype (n = 63; A/A). Log-rank test: *p *= 0.02. (*d*) Cumulative survival of ER-positive patients by A1298C status (n = 143). The survival of patients who carry the variant A1298C genotype (n = 70) is not significantly different from the survival of patients with the common genotype (n = 73). Log-rank test: *p *= 0.68.

We first examined the individual effects of the two genotypes on breast cancer survival assuming a dominant effect of the variant allele. C677T was not significantly associated with breast cancer survival in the unstratified Cox regression analysis with adjustments for age at diagnosis, race, A1298C, body mass index (BMI), ER status, TNM stage, and chemotherapy (Table 4). Stratification by ER-status and race revealed that the T allele (C/T or T/T) was protective in patients with an ER-negative tumor and in African-American patients. The association with ER-negative tumors was marginally statistically significant. The relationship between C677T and ER-negative but not ER-positive breast cancer was also seen in the Kaplan-Meier survival analysis (Figure 1a,b). To examine the independent effect of C677T, we repeated our analysis but included only those patients who were homozygous A/A at codon 1298 (n = 135, see Table 3). In this stratified analysis, the variant T allele at codon 677 (C/T or T/T) remained protective, albeit non-significantly, in patients with an ER-negative tumor (HR = 0.3; 95% CI, 0.06–1.47; n = 51) and in African-American patients (HR = 0.14; 95% CI, 0.02–1.09; n = 87).

The second *MTHFR *genotype, A1298C, was significantly associated with survival in the unstratified analysis (Table 5). In contrast to C677T, the variant allele (A/C or C/C) increased the risk of poor outcome. A stratification by the ER status showed that the variant C allele lead to an increased risk of dying only among patients with an ER-negative tumor, as shown in the Kaplan-Meier (Fig. 1c,d) and multivariate Cox regression analyses (Table 5). The variant C allele was also associated with a significantly increased risk of poor outcome among Caucasians and among patients, who did not receive chemotherapy (Table 5). To examine the independent effect of A1298C, we repeated our analysis but included only those patients who were homozygous C/C at codon 677 (n = 163, see Table 3). We found that the variant C allele at codon 1298 (A/C or C/C) remained a significant predictor of poor survival among ER-negative patients matched on the C677T genotype (HR = 3.27; 95% CI, 1.27–8.46; n = 62).

We also modeled the survival analysis assuming an additive effect (homozygous wild-type, heterozygous, homozygous variant) by C677T and A1298C. Again, we found that 677T was associated with an increased survival of patients who either had an ER-negative tumor (HR = 0.36; 95% CI, 0.13–1.02) or were African-Americans (HR = 0.12; 95% CI, 0.02–0.92). 1298C was associated with a decreased survival of patients with an ER-negative tumor (HR = 2.26; 95% CI, 1.17–4.37), Caucasians (HR = 2.53; 95% CI, 0.95–6.71), and patients who did not receive chemotherapy (HR = 2.74; 95% CI, 0.79–9.5). The results are very similar to those obtained with the assumption of a dominant effect by 1298C (Table 5). Thus, the additive and dominant models support the same conclusions.

### *MTHFR *diplotypes and breast cancer survival

Next, we studied the combined effect of the two SNPs after categorizing the genotypes into low and high MTHFR enzyme activity diplotypes based on the previously reported effect of the two variants on MTHFR activity [6]. Patients were classified as carriers of a high activity diplotype (n = 143) if they either had the CC (677) and AA (1298) or the CC (677) and AC (1298) genotype combinations (see Table 3). The CC(677)AC(1298) diplotype has 80–90% of the enzyme activity encoded by the CC(677)AA(1298) diplotype. All other genotype combinations were considered low activity diplotypes (n = 100) with a predicted MTHFR activity of 30–70% of the CC(677)AA(1298) diplotype. Carriers of a low activity diplotype had a non-significantly increased survival in the unstratified analysis and had a significantly increased survival if they were African-Americans (Table 6). Overall, the observed hazard ratios for the diplotypes were similar to the hazard ratios obtained for C677T but divergent from the hazard ratios obtained for A1298C.

### Interaction analysis

Lastly, we examined interactions between the SNPs and other risk factors on breast cancer survival. We did not observe an interaction with the ER status and chemotherapy. We found a significant protective interaction between the variant C allele at codon 1298 (A/C or C/C) and alcohol consumption on breast cancer survival (A1298C, *p*_*interaction *_= 0.017). Patients who reported to consume alcohol at recruitment had an improved survival, compared to nondrinkers, if they carried the 1298C allele because of the interaction. There was no such interaction with C677T or the *MTHFR *diplotype (C677T, *p*_*interaction *_= 0.43; *MTHFR *diplotype, *p*_*interaction *_= 0.64). We also observed interactions between the two genotypes and race/ethnicity on breast cancer survival (C677T, *p*_*interaction *_= 0.026; A1298C, *p*_*interaction *_= 0.088; *MTHFR *diplotype, *p*_*interaction *_= 0.054). Because of the interaction, African-American women had improved survival, compared to Caucasian women, if they were carriers of either the variant T allele at codon 677 (C/T or T/T), the variant C allele at codon 1298 (A/C or C/C), or the low activity *MTHFR *diplotype. The data are consistent with the results of the Cox regression analyses for C677T, A1298C, and the *MTHFR *diplotypes that showed an increased survival hazard of Caucasian women when compared with African-American women in the stratified analyses (Tables 4 to 6).

## Discussion

In our study of 248 African-American and Caucasian women with incident breast cancer, the *MTHFR *gene polymorphisms, C677T and A1298C, were associated with breast cancer survival. The effects were stronger in patients with estrogen receptor-negative tumors. We also observed an interaction between the two SNPs and race/ethnicity on breast cancer survival. The interaction decreased the survival hazard among African-American women and increased the survival hazard among Caucasian women. This finding indicates that race/ethnicity is an important modifier of the effect that *MTHFR *genotypes have on breast cancer survival.

It is plausible that *MTHFR *gene polymorphisms influence breast cancer survival. Folate is critical for DNA synthesis, DNA repair and epigenetic regulation of transcription. Folate deficiency causes uracil misincorporation into human DNA and induces chromosome breakage, and it has been argued that the protective effect of the 677T allele in colon cancer is related to a decreased incorporation of uracil into DNA [33,34]. There is evidence that the 677T allele increases thymidylate synthase activity in cancer cells because of an increased supply of 5,10-methyleneTHF, the methyl donor for methylation of dUMP to dTMP [8]. This increase of dTMP synthesis may come at the cost of a diminished global DNA methylation because cancer patients with a 677T allele (C/T or T/T) have constitutively low levels of 5-methylcytosine in their tumors and surrounding normal tissue [10]. Animal experiments support the notion that a low MTHFR activity leads to decreased DNA methylation. Heterozygous and homozygous *MTHFR *knockout mice have significantly lower S-adenosylmethione levels and global hypomethylation when compared to the *MTHFR *wild-type littermates [35]. Distinct DNA hypermethylation profiles have been associated with breast cancer and may lead to disease progression and metastasis [36]. However, *MTHFR *gene polymorphisms also affect the efficacy and toxicity of chemotherapeutics [37]. Because the direction of the effect is not uniform [19,20,38], and because intrinsic factors and lifestyle may further modify the effect of *MTHFR *gene polymorphisms, it is difficult to predict how *MTHFR *gene polymorphisms will influence cancer outcome.

There has been only one other study that examined the effect of *MTHFR *genotypes on breast cancer survival, and the authors did not observe a significant association between the two SNPs and survival of breast cancer patients from the Shanghai area in China [39]. Differences between these results and our associations could be due to examining different populations. Our study provided evidence that the effect of *MTHFR *genotypes on breast cancer survival differs by race/ethnicity. It is likely that the prevalence of factors that may influence the effect of *MTHFR *genotypes on breast cancer survival, such as tumor ER status, mode of therapy, or folate status, is different from patient population to patient population. For example, ER-negative tumors are more common among African-American women than other race/ethnic groups and these tumors are treated differently than ER-positive tumors [40]. Most patients in the Shanghai Breast Cancer study appear to have received 5-fluorouracil, a therapy that was infrequent in our patient group. Race/ethnicity is also a determinant of the folate status. African-Americans tend to have a lower folate intake than other race/ethnic groups and in a controlled trial, blood folate concentrations and folate excretion varied by race/ethnicity although the dietary folate intake and the C677T genotype were the same among all participants [26,27]. This observation that the folate status is different by race/ethnicity in a population matched on the C677T genotype (all C/C) and with identical folate intake may explain why we observed an interaction between race/ethnicity and the *MTHFR *genotypes on breast cancer survival. Finally, it should be mentioned that an ER-negative tumor status was a significant independent predictor of poor outcome in our population but it was not in the Shanghai Breast Cancer Study as judged by the 5-year survival rate [39].

The finding that *MTHFR *genotypes are associated with breast cancer survival depending on the tumor ER status is novel. It has been observed that total folate intake is associated with the incidence of breast cancer depending on the tumor ER status. In the Nurses' Heath Study, a higher folate intake was associated with a lower risk of ER-negative but not ER-positive breast cancer [17]. The authors hypothesized that adequate folate intake may be primarily important in preventing ER-negative tumors. ER-negative tumors may represent a distinct subtype of breast cancer, as revealed by the gene expression patterns of human breast tumors [41,42], and MTHFR may have a more significant role in the development and progression of this tumor type than ER-positive tumors. It is also possible that the estrogen receptor regulates the intracellular folate metabolism, a function, which would be lost in ER-negative tumors. There is support for this hypothesis. The estrogen receptor has been shown to repress the expression of the folate receptor α, and an inverse correlation between ER and folate receptor expression in breast cancer has been reported [24,25]. The folate receptor α is overexpressed in many human malignancies and shuttles folate, folate conjugates, and antifolate compounds into cells [43].

We found an opposite effect by C677T and A1298C on breast cancer survival although both variant alleles were found to decrease MTHFR enzyme activity [5-7]. The 677T variant allele increased survival among ER-negative patients. The 1298C variant allele was associated with a significantly decreased survival of ER-negative patients. There is evidence that the two SNPs may have different effects on the biochemical properties of MTHFR [9]. The A1298C leads to a substitution of a glutamine by alanine in the regulatory domain of the enzyme but does not cause thermolability of the enzyme or enzyme instability in the presence of low folate, like C677T [7,9]. Several reports showed that the two SNPs have different effects on the efficacy and toxicity of methotrexate and 5-fluorouracil. The 1298C variant allele was found to be associated with fewer adverse effects and higher efficacy and the 677T variant allele with an increased systemic toxicity in rheumatoid arthritis and cancer patients [20,21,23,38]. A methotrexate/5-fluorouracil-based therapy, however, was not a factor in our study. Most of the chemotherapy-treated patients received a combination of adriamycin and cyclophosphamide and only a few patients were treated with methotrexate and/or 5-fluorouracil.

Other studies observed opposite effects by these two SNPs. In the Long Island Breast Cancer Study, the 677T variant allele was associated with an increased risk of breast cancer and the 1298C variant allele was associated with a decreased risk of breast cancer [15]. The authors hypothesized that the inverse correlations were caused by the linkage disequilibrium between C677T and A1298C that links a low activity genotype at one locus to a high activity genotype at the other locus [31,32]. Even though we observed a linkage between the two SNPs in our study population, it does not fully explain the opposite effects of the two SNPs on survival. We performed additional analyses where we controlled for the confounding effect of the second *MTHFR *genotype. When we used this stratified approach, the two SNPs still had the same opposite effect on breast cancer survival. We further addressed the relationship between survival and *MTHFR *genotypes by categorizing patients into carriers of diplotypes that encode a MTHFR enzyme with either a low or high activity, as estimated *in vitro *[6]. In this analysis, which examines the effect of MTHFR activity rather than genotypes, the risk estimates for breast cancer survival were remarkably similar for the low activity diplotype and the low activity 677T variant but different from 1298C suggesting that the effect of C677T on survival mirrors the enzyme activity loss while the effect of A1298C does not.

Our study has several limitations. The current sample size did not allow us a more in-depth examination of the effect of race/ethnicity in a stratified analysis and we performed a number of subgroup analyses, which increases the possibility of chance finding. The subgroup analyses addressed hypotheses based on previous findings from observational studies suggesting that the ER-status, race/ethnicity, and chemotherapy could act as effect modifiers of the *MTHFR *genotypes. These hypotheses were formulated *a priori*, and our data that the ER-status and race/ethnicity modify the effect of *MTHFR *genotypes on survival are consistent with previous observations and biologically plausible. It is also a limitation that we could not include the patients' folate status into our analysis. Blood folate concentrations or a long-term assessment of the folate intake were not available. Folate intake is associated with the incidence of breast cancer and interactions between folate and *MTHFR *genotypes in relation to breast cancer risk have been observed in several epidemiological studies [2,14,15,17]. Alcohol acts as a folate antagonist and is a modifier of the risk association between folate and breast cancer [2,17,28]. We observed a significant interaction between A1298C and alcohol consumption on breast cancer survival. This observation may suggest that the folate status is a modifier of relationship between *MTHFR *genotypes and breast cancer survival. There is one report that examined the relationship between folate intake and survival of breast cancer patients with chemotherapy [44]. In the study, the authors did not observe a significant association between folate intake and breast cancer survival but the study did not examine *MTHFR *genotypes.

## Conclusion

We found that two functional polymorphisms in the *MTHFR *gene affect the survival of breast cancer patients with ER-negative tumors. We did not observe significant interactions between the two SNPs and chemotherapy on breast cancer survival but observed interactions with race/ethnicity and alcohol consumption.

## Abbreviations

MTHFR, 5,10-methylenetetrahydrofolate reductase; SNP, single nucleotide polymorphism; ER, estrogen receptor-α; HR, hazard ratio; BMI, body mass index.

## Competing interests

The author(s) declare that they have no competing interests.

## Authors' contributions

DNM and SA drafted the manuscript. DNM, BJB, and SA contributed to the design of the study. TMH, BJB, and SC contributed to data collection, DNA isolation, and genotyping. JEG, DNM, and SA performed statistical analyses. LEM and SC contributed to data analysis and interpretation. All authors read and approved the final manuscript.

## Pre-publication history

The pre-publication history for this paper can be accessed here:



## Figures and Tables

**Table 1:** Features of the breast cancer cases^1^

Age at diagnosis (mean ± SD; n = 248)	55.0 ± 13.9
Body mass index (mean ± SD; n = 248)	29.1 ± 8.0
	N (%)
Race/Ethnicity	
African-American	143 (58)
Caucasian	105 (42)
Menopausal status at diagnosis	
Pre-menopausal	64 (31)
Post-menopausal	141 (69)
Alcohol consumption	
No	93 (41)
Yes	133 (59)
Survival	
Alive	189 (76)
Deceased^2^	59 (24)
Tumor histology	
Ductal	189 (76)
Lobular	34 (14)
Others	25 (10)
Node status	
Negative	144 (63)
Positive	84 (37)
Stage at diagnosis (TNM)	
≤ Stage I	66 (29)
Stage II	118 (52)
≥ Stage III	44 (19)
Chemotherapy	
No	99 (43)
Yes^3^	132 (57)
Neoadjuvant therapy	
No	178 (77)
Yes	53 (23)
Estrogen receptor-α	
Negative	102 (41)
Positive	145 (59)
HER2/neu	
Low to weak	154 (62)
Moderate to strong	93 (38)

^1 ^Cases with missing information are not included. SD = standard deviation. Race/ethnicity is determined by self-identification. Pre-menopausal: still having menstruation. Post-menopausal: 55 years or older, or stated change of life, or hysterectomy with both ovaries removed. Alcohol consumption: yes if > 12 drinks in life at time of recruitment. ^2 ^For 5 patients, the cause of death was not breast cancer. ^3 ^Includes neoadjuvant therapy

**Table 2:** Distribution of C677T and A1298C genotypes by race/ethnicity^1^

	African-American n (%)	Caucasian n (%)	*p*-value^2^	Combined n (%)
C677T				
C/C	114 (81)	49 (48)	< 0.001	163 (67)
C/T	27 (19)	44 (43)		71 (29)
T/T	0	9 (9)		9 (4)

A1298C				
A/A	98 (69)	37 (36)	< 0.001	135 (56)
A/C	38 (27)	49 (48)		87 (36)
C/C	5 (4)	16 (16)		21 (8)

^1 ^Only cases who were successfully genotyped for both SNPs (n = 243) are included

^2 ^Chi-square test comparing genotype distributions between African-Americans and Caucasians

**Table 3:** Linkage between C677T and A1298C genotypes in the patient population

	C677T			
A1298C	C/C	C/T	T/T	Total

A/A	87	40	8	135
A/C	56	30	1	87
C/C	20	1	0	21
Total	163	71	9	243

**Table 4:** *MTHFR *C677T and breast cancer survival

	Person-years^1^	# events^2^	HR (95% CI)^3^	*p*-value
C677T genotype (n = 198)				
C/C			1	
C/T or T/T	873	46	0.65 (0.31–1.35)	0.25
ER-negative (n = 84)				
C/C			1	
C/T or T/T	352	29	0.36 (0.12–1.04)	0.06
ER-positive (n = 114)				
C/C			1	
C/T or T/T	521	17	1.12 (0.35–3.59)	0.82
African-American (n = 121)				
C/C			1	
C/T or T/T	520	29	0.12 (0.02–0.92)	0.04
Caucasian (n = 77)				
C/C			1	
C/T or T/T	352	17	1.89 (0.57–6.32)	0.3
No Chemotherapy (n = 81)				
C/C			1	
C/T or T/T	394	13	0.88 (0.21–3.74)	0.87
Chemotherapy (n = 117)				
C/C			1	
C/T or T/T	479	33	0.55 (0.22–1.34)	0.19

^1 ^Time at risk in person-years. ^2 ^Number of deaths due to breast cancer. ^3 ^Cox Proportional-Hazards regression with adjustments for age at diagnosis, race, A1298C, BMI, estrogen receptor, TNM stage, and chemotherapy.

**Table 5:** *MTHFR *A1298C and breast cancer survival

	Person-years^1^	# events^2^	HR (95% CI)^3^	*p*-value
A1298C genotype (n = 198)				
A/A			1	
A/C or C/C	873	46	2.05 (1.05–4.00)	0.03
ER-negative (n = 84)				
A/A			1	
A/C or C/C	352	29	2.70 (1.17–6.23)	0.02
ER-positive (n = 114)				
A/A			1	
A/C or C/C	521	17	1.39 (0.43–4.48)	0.58
African-American (n = 121)				
A/A			1	
A/C or C/C	520	29	1.57 (0.70–3.56)	0.28
Caucasian (n = 77)				
A/A			1	
A/C or C/C	352	17	6.28 (1.25–31.7)	0.03
No Chemotherapy (n = 81)				
A/A			1	
A/C or C/C	394	13	7.50 (1.49–37.7)	0.01
Chemotherapy (n = 117)				
A/A			1	
A/C or C/C	479	33	1.62 (0.76–3.46)	0.21

^1 ^Time at risk in person-years. ^2 ^Number of deaths due to breast cancer. ^3 ^Cox Proportional-Hazards regression with adjustments for age at diagnosis, race, C677T, BMI, estrogen receptor, TNM stage, and chemotherapy.

**Table 6:** *MTHFR *diplotypes and breast cancer survival

	HR (95% CI)^1^	*p*-value
*MTHFR *diplotypes (n = 198)^2^		
CC/AA + CC/AC (high enzyme activity)	1	
Others (low enzyme activity)	0.61 (0.3–1.22)	0.16
ER-negative (n = 84)		
CC/AA + CC/AC	1	
Others	0.47 (0.19–1.20)	0.11
ER-positive (n = 114)		
CC/AA + CC/AC	1	
Others C	0.83 (0.27–2.59)	0.75
African-American (n = 121)		
CC/AA + CC/AC	1	
Others	0.22 (0.05–0.96)	0.04
Caucasian (n = 77)		
CC/AA + CC/AC	1	
Others	1.19 (0.36–3.96)	0.78
No Chemotherapy (n = 81)		
CC/AA + CC/AC	1	
Others	0.54 (0.15–1.96)	0.35
Chemotherapy (n = 117)		
CC/AA + CC/AC	1	
Others	0.59 (0.25–1.38)	0.23

^1 ^Cox Proportional-Hazards regression with adjustments for age at diagnosis, race, BMI, estrogen receptor, TNM stage, and chemotherapy. Time at risk and number of events for the strata are shown in Tables 4 and 5.

^2 ^*MTHFR *diplotypes were generated from the C677T and A1298C genotypes using the information shown in Table 3. Diplotypes were dichotomized into a group that encodes a high activity enzyme [CC(677) and AA or AC(1298)] and a group that encodes a low activity enzyme (all other diplotypes).
